# Dihydropyrimidine Dehydrogenase Levels in Colorectal Cancer Cells Treated with a Combination of Heat Shock Protein 90 Inhibitor and Oxaliplatin or Capecitabine

**DOI:** 10.15171/apb.2019.052

**Published:** 2019-08-01

**Authors:** Mahshid Mohammadian, Shima Zeynali-Moghaddam, Mohammad Hassan Khadem Ansari, Yousef Rasmi, Anahita Fathi Azarbayjani, Fatemeh Kheradmand

**Affiliations:** ^1^Department of Clinical Biochemistry, School of Medicine, Urmia University of Medical sciences, Urmia, I.R. Iran.; ^2^Department of Pharmaceutics, School of Pharmacy, Urmia University of Medical sciences, Urmia, I.R. Iran.; ^3^Solid Tumor Research Center and Cellular and Molecular Research Center, Urmia University of Medical sciences, Urmia, I.R. Iran.

**Keywords:** Colorectal cancer, Oxaliplatin, Capecitabine, 17-AAG, Dihydropyrimidine dehydrogenase

## Abstract

***Purpose:*** Dihydropyrimidine dehydrogenase (DPD) is the principal enzyme in the catabolism of fluoropyrimidine drugs including capecitabine. A recent report has suggested that oxaliplatin chemotherapy is associated with elevated DPD levels and chemoresistance pattern. As a newly developed chemotherapeutic agent, 17-allyloamino-17-demethoxy-geldanamycin (17-AAG) can be effective in combination therapy with oxaliplatin and capecitabine in colorectal cancer (CRC). DPD expression level can be a predictive factor in oxaliplatin and capecitabine-based chemotherapy. We evaluated DPD in mRNA and protein levels with new treatments: 17-AAG in combination with oxaliplatin and capecitabine in HT-29 and HCT-116 cell lines.

***Methods:*** Drug sensitivity was determined by the water-soluble tetrazolium-1 assay in a previous survey. Then, we evaluated the expression levels of DPD and its relationship with the chemotherapy response in capecitabine, oxaliplatin, and 17-AAG treated cases in single and combination cases in two panels of CRC cell lines. DPD gene and protein expression levels were determined by real-time polymerase chain reaction and western blotting assay, respectively.

***Results:*** DPD gene expression levels insignificantly increased in single-treated cases versus untreated controls in both cell lines versus controls. Then, the capecitabine and oxaliplatin were added in double combinations, where DPD gene and protein expression increased in combination cases compared to pre-chemotherapy and single drug treatments.

***Conclusion:*** The elevated levels of cytotoxicity in more effective combinations could be related to a different mechanism apart from DPD mediating effects or high DPD level in the remaining resistance cells (drug-insensitive cells), which should be investigated in subsequent studies.

## Introduction

Colorectal cancer (CRC) is a common cancer with high rate of morbidity and mortality throughout the world. In chemotherapy, as a main method of cancer therapy, treatment involves administering pharmaceutical agents to destroy tumor cells.^1,2^ It has been shown that oxaliplatin and capecitabine monotherapy or as co-administration have acceptable effects on CRC in clinic^3,4^ ; however, problems including drug resistance and side effects introduce challenges to evaluate new combinations.^5,6^

Recently, heat shock protein 90 (HSP90) inhibitor agents including 17-allyloamino-17-demethoxy-geldanamycin (17-AAG), a geldanamycin analogue, has been developed as a novel cancer drug target. This drug is currently in phase II clinical trials for numbers of cancers and in some in vitro studies has been assayed in CRC.^7-17^ Cytotoxic effects of 17-AAG in combination with oxaliplatin, 5-fluorouracil (5-FU) and capecitabine, were reported in previous studies.^14-16^ In our previous study, 17-AAG revealed synergistic interaction with oxaliplatin and capecitabine in double combinations at the concentration of 0.5× IC50 in HCT-116 and HT-29 cell lines.^17^

In planning chemotherapeutic drugs, it is important to evaluate the cancer response against chemotherapy.^17^ The response rate of tumors to fluoropyrimidine drugs depends on thymidylate synthase and dihydropyrimidine dehydrogenase (DPD) activity.^18^ DPD is a main enzyme in the biochemical functions of the antimetabolite 5-FU as well as capecitabine.^19-21^

Indeed, DPD is considered as regulatory enzyme in the 5-FU catabolic pathway which converts 5-FU to 5-fluorodihydrouracil. Low DPD expression levels have proved to be related to altered catabolism of 5-FU and consequently further accumulation and better effect on tumor control. Instead, elevated DPD levels lead to drug resistance by reducing the cytotoxic effects of 5-FU.^22,23^

A previous study also reported increased levels of DPD after oxaliplatin treatment which has been associated with treatment resistance.^24^ Indeed, DPD dysregulation has been shown to be associated with the toxicity of these drugs.^20,24,25^

As combination of 17-AAG with oxaliplatin and capecitabine has proved to have a higher impact on tumor inhibition^17^ , in this study, we aimed to investigate the effects of these combinations on DPD gene and protein expression levels in the panel of two CRC cell lines (HT-29&HCT-116).

## Materials and Methods

### Cell lines and drug treatments

The human CRC cell lines HT-29 and HCT-116 were obtained from Pasteur institute (Iran, Tehran) and maintained according to the instructions provided by the American Type Culture Collection. Cell culture materials were purchased from Biowest (France). Capecitabine, oxaliplatin and 17-AAG were obtained from Sigma-Aldrich (USA) and LC Corporation (USA) respectively. Stock solution of each drug was prepared in water at the concentration of 10mg/ml (capecitabine and oxaliplatin) and 50 μg/mL (17-AAG). The drugs’ effects were evaluated based on water-soluble tetrazolium-1 (WST-1) assay in different concentrations to get IC50 values according to Chou and Talalay,^26,27^ method mentioned in our previous work.^17^

The cytotoxic effects of each single drug examined at different concentrations including 0.5, 1, 2, 4, and 8 μM for capecitabine and oxaliplatin, and 0.005, 0.01, 0.020, 0.04 and 0.08 μM for 17AAG for 24 hours. Double-combination treatments (capecitabine and oxaliplatin, capecitabine and 17-AAG, oxaliplatin and 17-AAG) examined at 2 × IC_50_, 1 × IC_50_, 0.5 × IC_50_, and 0.25 × IC_50_ concentrations in both cell lines for 24 hours.

Drug dosages for single treatments and double combinations were selected according to WST-1 analysis (IC50 for single drug and 0.5× IC50 for double drug combination), which has been performed in the previous study.^17^

### Real-time polymerase chain reaction (PCR) analysis

For extracting total RNA, about 10^7^of HT-29 and HCT-116 treated and untreated cells were harvested for 24 hours in 6-well plates.

The real time PCR examined at IC50 concentrations in single drug treatments after 24 hours. Also, double combination treatments (capecitabine and oxaliplatin, capecitabine and 17-AAG, oxaliplatin and 17-AAG) tested at 0.5 × IC_50_ concentrations in both cell lines for 24 hours.

Afterwards the cells were trypsinized and total RNA was isolated using the RNA extraction kit according to the manufacturer’s protocol (GeneAll, South Korea) and the extracted RNA purity was evaluated by measuring the ratio of optical density at 260 nm to that at 280 nm. In addition, RNA integrity was assessed by agarose gel electrophoresis. First strand cDNA synthesis was synthesized using SuperScript III^™^ First Strand synthesis kit (GeneAll, South Korea). Then, real-time PCR was performed in a total volume of 25 µL using AccuPower^®^2× Green StarqPCR master mix (Ampliqon, Denmark) based on the manufacturer’s protocols. Real time-PCR using cDNAs and specific primers of DPD and β-Actin was performed at 30 cycles of denaturation for 30 s at 95°C, annealing for 30 seconds at 59°C, and extension for 30 seconds at 72°C. Primer sequences of β-actin and DPD genes were presented in Table 1.

**Table 1:** Sequences of primers used to evaluate the expression of β-actin and DPD genes in HT-29 and HCT-116 cell line

**Target Gene**		**Primer Sequence**	**Product Size**
β-actin	Forward	5´-CTGGAACGGTGAAGGTGACA-3´	161
	Reverse	5´-TGGGGTGGCTTTTAGGATGG-3´	
DPD	Forward	5'-CGGTGAATGATGGAAAGCAAG-3'	99
	Reverse	5'-AAAAGAGGGGTAGTTCAGGC-3'	

DPD; Dihydropyrimidine dehydrogenase.

A melting curve analysis was done to confirm the specificity of the amplification reactions. Each sample was replicated at least three times and the threshold cycle (Ct) values were evaluated. Finally, the relative expression of mRNA in the current study was calculated via the 2^-RΔΔCt^ method.^28^

### Western blotting

HT-29 and HCT-116 cells were treated with IC50 concentrations of each tested drug in single treatments and 0.5×IC50 in double combinations for 24 hours. Then, these cells were trypsinized and washed with PBS. Cell lysate was prepared by incubation of the cells with RIPA lysis buffer (Bio-Rad, USA) with protease inhibitor cocktail (Sigma, USA). Afterwards, cell lysate was centrifuged in 12000×g, 20 min in 4°C and supernatant was used for protein level (concentration) determination. Protein concentration was measured with a protein assay kit (Bio-Rad), with bovine serum albumin (Sigma-Aldrich) as a standard. 1000 μg of protein were utilized for electrophoresis on sodium dodecyl sulfate (SDS)–polyacrylamide gel. For loading samples on SDS-polyacrylamide gel, each specimen was incubated for 10 minutes at 65°C. After electrophoresis, the proteins were transferred onto a polyvinylidene difluoride membrane (Biorad, USA) in a transfer buffer. Nonspecific sites were blocked with 5% skim milk; then were incubated with primary anti-DPD and β-Actin mouse monoclonal antibodies overnight; the incubation with secondary antibodies linked to horseradish peroxidase (HRP) was done for three hours. Detection was carried out using a TMB stabilized substrate for HRP (Cytomatin Gene Co, Isfahan, Iran) according to the manufacturer’s protocol. Results were analyzed by ImageJ software version 1.49v (NIH). Band densities were normalized to β-actin protein expression.

### Statistical analysis

Statistical analysis was performed with GraphPad Prism software version 4.0 (GraphPad Software Inc., San Diego, CA). For measuring relative expression of mRNA, the 2^-RΔΔCt^method was utilized.^28,29^ Relative expression levels of mRNA were normalized to ß-actin and then were analyzed for statistical significance with one-way ANOVA method. A *P* value < 0.05 was considered statistically significant.

## Results and Discussion

### Effects of 17-AAG, oxaliplatin and capecitabine in single and double drug treatments on DPD gene expression

**Figure 1:**
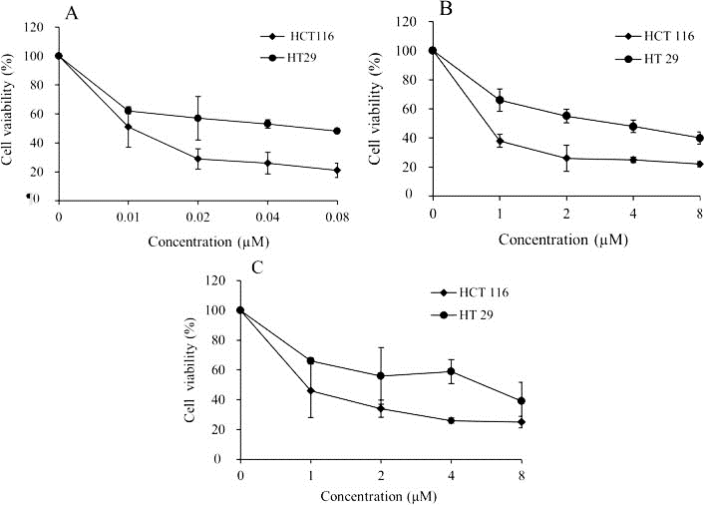
Cytotoxic effects of (A),17-AAG; (B), capecitabine, and (C), oxaliplatin in HT-29 and HCT-116 cell lines (With permission).^17^

According to our findings upon the previous WST-1 analysis,^17^ HT-29 cell line had higher IC50 values in the single drug treatments in compared to HCT-116 cells. The cytotoxic effects of the three examined drugs after 24 hours were presented in Figure 1 (with permission).

Data were shown as mean ± standard deviation. HT-29 and HCT-116 cell lines were treated with different concentrations of each drug. Cell viability in the treated cells was measured by WST-1 assay and was compared to untreated cells. 17-AAG: 17-Allylamino-17-demothoxygeldanamycin; WST-1; water-soluble tetrazolium-1 assay.

As double combinations (0.5×IC50 concentrations) of each drug had more effective growth inhibitory results in comparison to the higher doses of single drug treatments, we selected this concentration (0.5×IC50) for DPD level analysis in double combination groups (1.7 and 0.75µm for capecitabine, 1.9 and 0.75µm of oxaliplatin, 35 and 9.45 nm of 17-AAG for HT-29 and HCT-116, respectively).^17^

**Figure 2:**
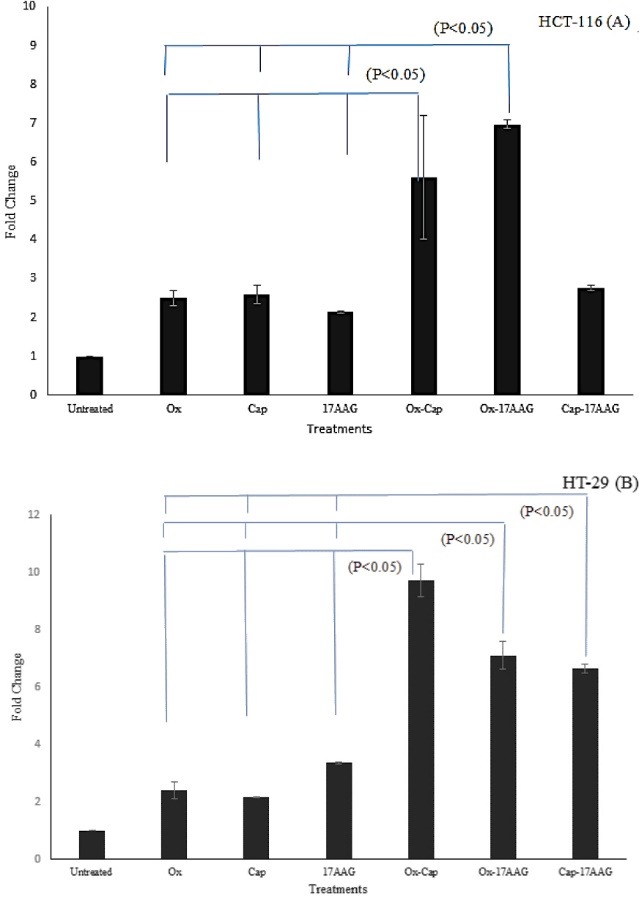
Real-time PCR analysis to determine the effects of 17-AAG, capecitabine and oxaliplatin in single (IC50 concentrations) and double combination (0.5×IC50 concentrations) treatments on DPD mRNA levels with β-Actin as an internal control in HCT-116(A) and HT-29 (B) cell lines. Vertical bars presented the mean fold change ± standard deviation for independent experiments of real-time PCR.
17AAG, 17-Allylamino-17-demothoxygeldanamycin; Ox, Oxaliplatin; Cap, Capecitabine; Real-time PCR, Real-time polymerase chain reaction.

According to our results, there were insignificant differences in DPD mRNA levels in IC50 doses of all single drug treatments (capecitabine, oxaliplatine and 17-AAG), compared to the control groups in both cell lines (*P* > 0.05). In double combination cases of HT-29 cell line, there were significant increase in DPD level compared to the single drug treatments (*P* < 0.05). In HCT-116 cell line, only oxaliplatin-capecitabine and oxaliplatin-17-AAG combinations had higher levels of DPD mRNA versus single drug treatments (*P* < 0.05; Figure 2).

### Effects of 17-AAG, oxaliplatin and capecitabine in single and double drug treatments on DPD protein levels

**Figure 3:**
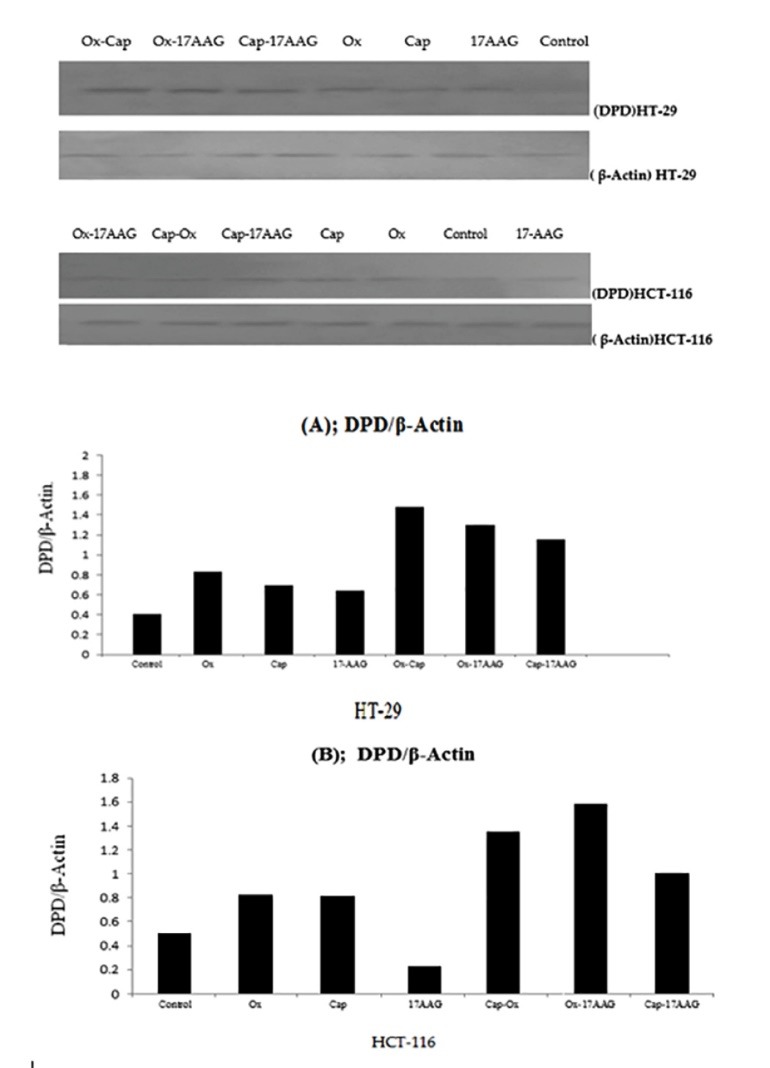
DPD protein expression of 17-AAG, capecitabine and oxaliplatin in single (IC50) and double (0.5× IC50) treatments in HT-29 (A) and HCT-116 (B) cells. After cell culture and drug treatments, total cell lysate was isolated and analyzed for DPD protein expression by western blotting method. The relative DPD protein levels determined by densitometric analysis of immunoblot and normalized to β-Actin. Ox; oxaliplatin; Cap, Capecitabine; 17AAG, 17-Allylamino-17-demothoxygeldanamycin; Ox, Oxaliplatin; Cap, Capecitabine.

Western blotting analysis (Figure 3) showed increased DPD protein levels in single drug treatments compared to untreated control groups in both cell lines (except 17-AAG in HCT-116). Among single treatment groups, oxaliplatin-treated cells had higher DPD levels versus other single treatments in both cell lines. 17-AAG in single drug treated cases had lower DPD protein expression in comparison with oxaliplatin and capecitabine in HCT-116 and HT-29. In double combinations, there were elevated levels of DPD compared to single drug treatments in both cell lines. In double drug combinations, oxaliplatin-capecitabine and oxaliplatin-17AAG combinations showed higher DPD protein levels versus other double combinations in HT-29 and HCT-116, respectively.

Although capecitabine is a major agent in combination therapy, there are no verified markers to predict the clinical outcome of capecitabine alone^30^ and in combination with other drugs in CRC.

A previous study indicated that the gene expressions of the pyrimidine metabolism enzymes including DPD are related to response determinants of fluoropyrimidine-based drugs in different tumor types.^30^ Also, elevated levels of DPD were reported after oxaliplatin therapy in CRC patients.^24^

DPD dysregulation has been shown to be involved in occurrence of the adverse events of fluoropyrimidine- and oxaliplatin treatments.^24,25^ In this study, the DPD levels was evaluated after treatments with capecitabine and oxaliplatin in combination with 17-AAG in CRC cells.

According to our results, DPD gene expression proved to be higher in HT-29 cells compared with HCT-116 cell line.

As elevated DPD levels lead to drug resistance,^22^ higher IC50 levels were observed for our examined drugs in HT-29 compared to HCT-116 (according to our previous work^17^ ), might be a sign of higher sensitivity of HCT-116 cell line to tested drugs. Nevertheless, based on WST-1 results, we obtained higher cytotoxicity in double combination compared to single drug treatments in both cell lines.^17^ There were significant increase in DPD mRNA levels in all double combinations (except cap-17-AAG in HCT-116). Protein expression levels by western blot analysis were parallel to mRNA gene expression results (increased partially) in both cell lines.

In this regards, Vallbohmer et al reported that patients with a lower level of DPD mRNA had a longer progression-free survival versus other patients with increased DPD mRNA level.^30^ Also, Baba et al reported that after oxaliplatin-based first-line chemotherapy, there were increased DPD expressions in metastatic CRC,^24^ suggesting greater drug resistance in tumor cells with higher DPD levels.

As colorectal tumors with good response to chemotherapy with 5-FU had low DPD gene expression levels,^31^ the higher cytotoxicity levels in our double treated groups might be a sign of involvement of some other pathways (except DPD pathway) like apoptosis or oxidative stress on the effect of the combination of chemotherapeutic agents as compared to single group drugs.^14-16^

In another study, Murakawa et al studied the clinical implications of patients with pancreatic cancer undergoing curative resection with oral 5-FU prodrug tegafur combined with oteracil and gimeracil. They reported that there was a significant difference in the 3-year overall survival rates after surgery in the DPD-high as compared to DPD-low expression patients.^32^

In addition, in another survey by Yoshida et al, elevation of DPD protein levels has been reported^33^ (approximately 12-fold compared to before chemotherapy) after capecitabine dose increase in combination with oxaliplatin and bevacizumab.

As in HCT-116 cells treated cells with 17-AAG-capecitabine, the level of DPD was very low (as much as most single treated groups); it seems that this combination might have a better response in the treatment of CRC.

Accordingly, in the study by Zeynali-Moghaddam et al, this combination revealed a better response in terms of angiogenesis and cytotoxicity in HT-29 cells.^16^ However, as low DPD is associated with elevated toxicity in cancerous patients,^34^ the clinical efficacy of this combination regarding possible side effects should be studied further.

On the other hand, the other probable mechanism related to elevated levels of DPD in double combinations may be related to high DPD levels in a minor percentage of cancer stem cells, which may remain after destroying drug-sensitive cells by chemotherapy according to Baba et al.^24^ Then, long-term follow-up of the effect of double combination treatments on cell lines and in animal studies could be helpful to discover the relevant causes of increased double combination cases.

## Conclusion

Chemotherapy resistance remains one of the greatest challenges in metastatic cancers. Nevertheless, chemotherapeutic agents, which effectively inhibits uncontrolled proliferation of cancerous cells and induce cell death, are prominent candidates for development. So, it is important to improve the treatment outcome by assessing cancer response.^17,35,36^ DPD is an important enzyme in the biochemical functions of the antimetabolite drugs whose altered expression is related to adverse events following fluoropyrimidine- and oxaliplatin-based treatments.^24,25^ In two panels of CRC cell lines, double chemotherapy with capecitabine, oxaliplatin, and 17-AAG was superior to single chemotherapy in terms of efficacy.^17^ The elevated levels of cytotoxicity in more effective combinations could be related to different mechanisms apart from DPD mediating effects in double combinations.

As DPD expression level was inversely associated with chemosensitivity,^37^ the other explanation may be attributed to high DPD levels in the remaining resistance cells (drug-insensitive cells). Further studies could be conducted to evaluate the molecular mechanisms in drug resistance pathways in relation to DPD gene and protein expression pattern.

## Ethical Issues

This article does not involve any studies with human or animals subjects.

## Conflict of Interest

None.
